# Mechano-adaptation of the stem cell nucleus

**DOI:** 10.1080/19491034.2017.1371398

**Published:** 2017-11-13

**Authors:** Su-Jin Heo, Brian D. Cosgrove, Eric N. Dai, Robert L. Mauck

**Affiliations:** a McKay Orthopaedic Research Laboratory, Department of Orthopaedic Surgery, Perelman School of Medicine, University of Pennsylvania, Philadelphia, PA, USA; b Department of Bioengineering, School of Engineering and Applied Science, University of Pennsylvania, Philadelphia, PA, USA

**Keywords:** Epigenetics, Heterochromatin, Lamin A/C, LINC Complex, Mechanotransduction, Nuclear Mechanics, Stem Cells

## Abstract

Exogenous mechanical forces are transmitted through the cell and to the nucleus, initiating mechanotransductive signaling cascades with profound effects on cellular function and stem cell fate. A growing body of evidence has shown that the force sensing and force-responsive elements of the nucleus adapt to these mechanotransductive events, tuning their response to future mechanical input. The mechanisms underlying this “mechano-adaptation” are only just beginning to be elucidated, and it remains poorly understood how these components act and adapt in tandem to drive stem cell differentiation. Here, we review the evidence on how the stem cell nucleus responds and adapts to physical forces, and provide a perspective on how this mechano-adaptation may function to drive and enforce stem cell differentiation.

## Overview

Mechanical forces play a key role in numerous cellular processes, including adhesion, migration, and differentiation and, at the organismal level, direct tissue development, morphogenesis, and regeneration. In the 1990′s, Maniotis and colleagues first demonstrated that an exogenous mechanical force applied to a cell could result in nuclear deformation, leading to the hypothesis that mechanical forces could directly regulate gene expression.^1^ Two decades later, new technologies enabled the demonstration, for the first time, that physical forces acting at the cell boundary and through the cytoskeleton can indeed reposition chromatin segments and alter gene expression over very short timescales.^2^ In order for this mechanotransduction event to occur, exogenous force must first be translated through cytoskeletal elements that physically connect the nucleus to its environment. Over the past decade, these cytoskeletal-to-nuclear connections and structures have been increasingly well defined and their role in mechanotransduction demonstrated.^3-5^ Indeed, a growing body of literature now suggests these elements are not only required for changes in mechanically activated signaling and changes in gene expression,^2,3^ but also that they are dynamically adaptive, with aspects like nuclear structure, connectivity, and reinforcement changing in response to mechanical loading.^6^ Likewise, it has been shown that the nucleus itself is not only the stiffest organelle in the cell, but that its internal structure (and mechanical properties) can adapt over short and long time scales in response to mechanical perturbation.^4,7,8^ Thus, multiple dynamic components act cooperatively to regulate the mechanical state of the nucleus and gene expression, which in turn feeds back through nascent protein production to inform and update the mechanical state of the whole cell.

**Figure 1.**
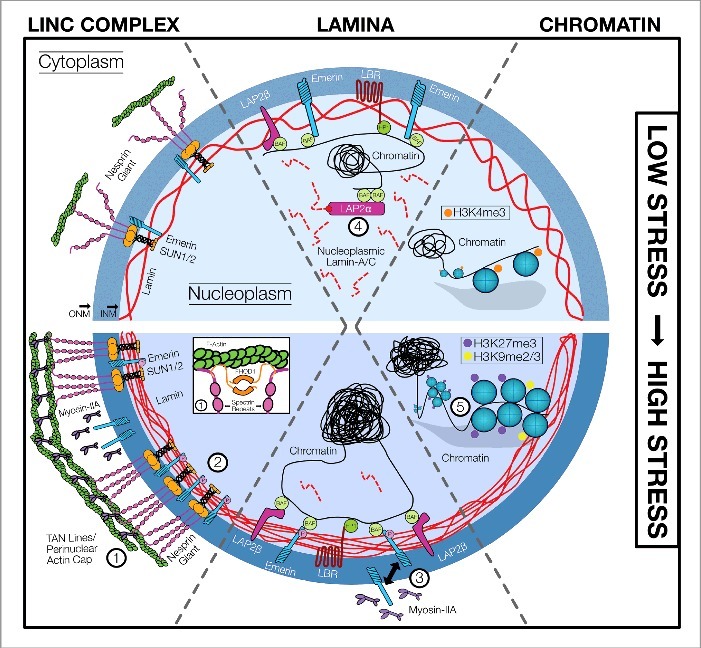
Schematic representation of mechano-adaptation in multiple compartments of the stem cell nucleus. *Left*: The LINC complex spans the nuclear membrane, mechanically linking the cytoskeleton to the nucleus and sub-nuclear structures. Nesprin giant isoforms cross the nuclear membrane, binding to F-actin and other cytoskeletal elements in the cytosol and to SUN proteins in the intra-nuclear space. SUN proteins in turn tether nesprins to the nuclear lamina. The LINC complex responds dynamically and adapts to changing stress within the cell. In low stress states, emerin closely associates with SUN at the INM, and nesprins form minimal contacts with the cytoskeleton. Under high stress conditions, nesprins cluster and are under tension (1), forming characteristic features known as ‘TAN lines' across the apical side of the nucleus. Further, emerin undergoes tyrosine phosphorylation (2), with a fraction of this protein translocating from the inner nuclear membrane (INM) to the outer nuclear membrane (ONM) in the high stress state, where it helps to locally increase the Myosin-IIA concentration (3). *Middle*: The nuclear lamina is composed of a meshwork of filamentous lamins that are central in the establishment of nuclear structure and mechanics. BAF binds to emerin at the INM, and also functions to tether nucleoplasmic LAP2α to chromatin. There is a balance of soluble nucleoplasmic lamin-A/C and stable lamin-A/C that is juxtaposed to the INM in a network (4). In all states, LAP2β localizes to the INM, and along with emerin, tethers chromatin to the lamina through interactions with BAF. LBR likewise interacts with HP1 to localize chromatin to the lamina. In high stress states, the pool of nucleoplasmic lamin-A/C is reduced as lamin-A/C translocates to the lamina, where it is assembled into a denser network that mechanically reinforces and stiffens the nucleus overall. *Right*: In addition to its role in storing genetic information, chromatin is also a critical determinant of nuclear mechanics. Under low stress conditions, the global chromatin state is generally open and active, with histones modified with active marks, including H3K4me3; additionally, the nucleus is relatively soft, due in part to the de-condensed state of the chromatin. Under high stress conditions, conversely, chromatin condenses and becomes transcriptionally repressed on a global scale (5). This results in nuclear stiffening and a general enrichment of repressive histone marks, including H3K27me3 and H3K9me2/3.

**Figure 2.**
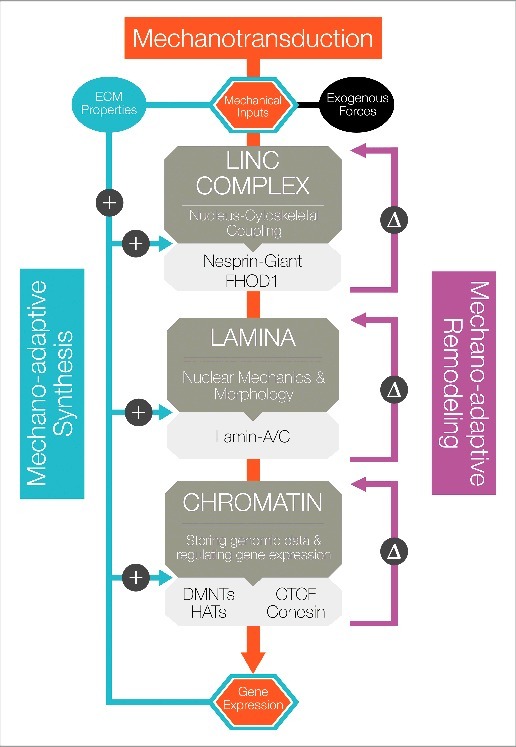
Mechano-Adaptation and Mechanical Memory Nuclear mechano-adaptation in stem cells can occur through both remodeling and synthetic mechanisms, with the former occurring over faster time scales than the latter. Mechanical inputs from the ECM or from exogenous loading are transmitted through the cell and to the nucleus, where each compartment can undergo mechano-adaptation, depending on the magnitude and repetition of the mechanical cues. The LINC complex responds and adapts through increased clustering of nesprins and TAN line formation (remodeling), as well as increased production of components of the LINC complex (synthesis). The lamina undergoes stiffening via reinforcement of the lamin meshwork from the nucleoplasmic pool of soluble lamin-A/C, as well as increased production of lamin-A/C. Finally, the chromatin adapts through marked changes in spatial organization, as indicated by increased condensation levels, as well as increased transcription of proteins associated with chromatin structural reinforcement and stabilization. More broadly, synthetic and reorganizational changes combine to alter the properties of each nuclear compartment, such that the next mechanical input encountered by the cell is transduced to and through the nucleus in a slightly different manner. This mechanically mediated reconfiguration of the LINC complex, lamina, and epigenome alters overall cell mechanosensing and may confer a ‘mechanical memory’ in the system.

This mechano-adaptive process is often initiated by the dynamic remodeling of a protein or complex of proteins in response to applied force, due to either a direct physical change in structure or complex organization or through a physically-induced signaling pathway that elicits the same change. In focal adhesions, for example, growth and/or shrinking arise as a consequence of force in the actin cytoskeleton causing force-induced unfolding, revealing cryptic binding domains that enable assembly of larger structures.^9^ Similar force-induced unfolding events have also been reported in cadherin-based cell-cell adhesions.^10,11^ Additionally, mechano-adapation can occur through the synthesis of new proteins that act to reinforce and initiate mechanotransductive signaling pathways. The functional implications of these mechano-adaptive processes are prominent across a range of cellular contexts, especially during stem cell differentiation. In this article, we review evidence related to how components of the stem cell nuclear force sensing machinery undergo mechano-adaptation in response to exogenous forces (Fig. 1), and importantly, how this dynamic feedback might both inform and enforce lineage specification in stem cells (Fig. 2). This perspective will focus on mechano-adaptation in three distinct compartments: 1) the connection between the cytoskeleton and the nucleus (the LINC complex), 2) the nuclear lamina, 3) and the epigenome (including the lamina-to-chromatin interface and the chromatin itself).

## Nuclear structure, connectivity, and the epigenome

The nucleus is the largest organelle and signaling center of the cell and contains the majority of the cellular genetic material. Notably, the nucleus houses chromatin, the complex of DNA with histones and structural proteins (lamins and other nucleoskeletal proteins) that help to establish nuclear shape and mechanics. The collective composition and arrangement of chromatin is referred to as the epigenome. At the inner nuclear envelope, a specialized set of proteins, the type-V intermediate filament lamins, associate to form the nuclear lamina, which functions to reinforce the nuclear envelope^12^ and can act to modulate transcription.^13^ Lamins are divided into two main subtypes: lamin-A/C and lamin-B1/B2. While B-type lamins are present in all cells, A-type lamins are expressed only in differentiated cells, where they form mesh network on the inner nuclear membrane (INM) and are present in the nucleoplasm in soluble form.^14^

The nucleus (and its contents) is also physically tethered to the cytoskeleton through a protein complex known as the Linker of Nucleus and Cytoskeleton (LINC) complex. Compared to the actin cytoskeleton and its connections to the external environment via membrane based adhesion complexes, less is known about the LINC complex. Two principal components, nesprins and SUN proteins, span the nuclear envelope and connect the nuclear lamina to the actin cytoskeleton. Due to alternative splicing, there are many nesprin protein variants, each with distinct nuclear connectivity and cellular distribution; the largest isoforms are dubbed the “giant” nesprins due to their ∼1 MDa size. Additionally, the N-terminus of the giant isoforms of Nesprin-1 and −2 contain a calponin homology (CH) domain that links to F-actin. The C-terminus of these giant nesprin isoforms contains a KASH domain, which passes through the outer nuclear membrane and interacts with SUN proteins in the inner nuclear membrane. Other nesprin isoforms bind to alternate cytoskeletal components, including microtubules via kinesin-1 (Nesprin-4) and intermediate filaments via plectin (Nesprin-3).^15^ SUN proteins reside in the perinuclear space and span the inner nuclear membrane, where their N-termini interact with the nuclear lamina (including lamin-A/C as well as lamins-B1 and -B2). This membrane spanning interaction allows for physical anchorage of these complexes, enabling exogenous force transmission through the cytoskeleton to the nuclear lamina. SUN proteins can additionally associate with nuclear pore complexes,^16^ emerin,^17^ and other inner nuclear membrane (INM) proteins.^18^ Proper strain transfer to the nucleus and subsequent nuclear remodeling in response to exogenous force depends on this physical linkage,^3,4,19^ as will be described in further detail below.

## Mechano-adaptation of the linc complex

Nuclear connectivity through the LINC complex is essential for many cell functions, including development and differentiation,^20^ and likewise plays an important role in disease and aging.^21^ Given the similarity between LINC complexes and transmembrane adhesive complexes, such as focal adhesions (mediating cell-ECM interactions) and adherens junctions (mediating cell-cell interactions), many have speculated that higher-order LINC complex structures exist at the nuclear boundary, and that these may respond to changing mechanical inputs (Fig. 1*; LINC Complex*). Corroborating this speculation, Gundersen and colleagues showed that clusters of LINC complexes are enriched directly beneath apical stress fibers running over the nucleus; they dubbed these structures ‘TAN lines’ (for ‘Transmembrane Actin-Associated Nuclear lines’).^22-24^

The notion of mechanical force transmission through nesprins was recently supported by the development of a mini-Nesprin-2G isoform (lacking some of the spectrin repeats) engineered to act as a FRET-based tension sensor. Studies using this molecule showed that nesprins are indeed under tension, which would potentially allow for participation of nesprins in many previously elucidated force-induced signaling mechanisms.^25^ Combining micropatterning approaches with this tension sensor further revealed that the LINC complex experiences increased loads as cytoskeletal force increases. However, given that this construct lacked spectrin repeats normally present in the full length molecule, additional work will be required to determine how the native protein operates and remodels under changing load.^26^ Mechano-adaptation at this LINC complex might also be achieved by additional protein interactions, for instance with FHOD1, an actin-bundling formin. This molecule has binding domains for both nesprin and actin, and could function to organize or reinforce LINC complexes under load.^23^ In fact, FHOD1 interactions (in 2D) are required for TAN line formation with changing boundary conditions (e.g. in a scratch assay). Adding to this complexity, the inner nuclear membrane (INM) protein Samp1 is also enriched in TAN lines, and is required for proper TAN line formation due to its role in reinforcing the connection of SUN proteins with the nuclear lamina.^18^

Moving forward, additional studies will be required to map out the constituents of these nuclear-to-cytoskeletal adhesion complexes and how they mechano-adapt with applied force. Single molecule localization microscopy (SMLM) approaches have recently begun to provide additional insight into both focal adhesion and adherens junction organization, revealing novel paradigms of their force-driven reinforcement.^27,28^ Likewise, live cell imaging probes that report back on the stress in these molecules will be essential for probing the kinetics and response functions of mechano-adaptation in these systems. Similar approaches were central to our understanding of focal adhesion dynamics,^29^ though the large size of nesprin giant may make delivery and/or expression of full length molecules a greater challenge. Nevertheless, studies are beginning to establish the array of proteins in the LINC complex that regulate mechano-adaptation of this critical linkage of the nucleus to the cytoskeleton, bridging our understanding of how extracellular mechanical cues are transmitted to the nuclear interior.

## Mechano-adaptation of the nuclear lamina

While the LINC complex is responsible for transmitting cytoskeletal forces to the nucleus, the nuclear lamina itself plays a critical role in translating and adapting to these exogenous forces. Early studies revealed the central role of lamin-A/C in defining nuclear shape and stiffness, with knockdown of this molecule resulting in changes in nuclear morphology at baseline and increased nuclear deformation, defective signaling, and impaired viability when cells were exposed to mechanical perturbation.^30^ Recent work by Swift and colleagues showed that the state of the nuclear lamina adapts to its surrounding environment. More specifically, the ratio of lamin-A/C to lamin-B was found to scale with the surrounding tissue micro-elasticity, where the nuclei of cells situated within stiffer tissues contained relatively higher amounts of lamin-A/C compared to those in softer tissues.^7^ It has been suggested that the assembly-disassembly dynamics of lamins are mediated by force-induced changes in their conformation. For instance, lamin antibodies with different epitopes show changes in accessibility as a consequence of altered force generation within the cell.^31^ Additionally, force on lamin can lead to a conformational change that leads to accessibility of otherwise buried cysteine residues.^7^ Taken together, these studies suggest the existence of a mechano-active feedback loop at the nuclear lamina (Fig. 1*; Lamina*), where external mechanical inputs result in differential cytoskeletal pre-stress, altering lamin-A/C expression, assembly, and stability, and ultimately nuclear mechanics.^7^

Importantly, this adaptive regulation of lamin-A/C also plays a role in stem cell differentiation. One hallmark of embryonic stem cells is their lack of lamin-A/C.^32^ While only B-type lamins are found in undifferentiated mouse and human embryonic stem (ES) cells, differentiation is accompanied by the onset of A/C-type lamin expression.^33^ Indeed, early micropipette aspiration studies showed large, lamin-A/C dependent increases in nuclear stiffness as cells transitioned from an embryonic to a differentiated state.^34^ Lamin-A/C content can also regulate differentiation potential in stem cells; knockdown or overexpression of lamin-A/C in mesenchymal stem cells (MSCs) strongly modulates their adipogenic and osteogenic potential as well as their interpretation of physical cues from the microenvironment.^7^

Whereas expression-mediated alterations in lamin-A/C can change nuclear mechanics over hours to days, other mechanisms allow for a more rapid mechano-adaptation of the nuclear lamina. For example, Philip et al. reported changes in lamin-A concentration and organization in HeLa cells in response to fluid-induced shear stress.^8^ Buxboim and colleagues also showed dynamic remodeling and degradation of lamin-A/C under conditions of altered cell tension, and that the nucleoplasmic fraction of lamin-A/C exhibits greater mobility than that assembled into the cortical shell.^35^ Introduction of phospho-mutants of lamin-A/C revealed that specific residues control this complexation at the nuclear membrane^36^ and that nuclear stiffening occurs through increased incorporation of soluble nucleoplasmic lamin-A/C into the nuclear lamina. Cells in low-tension microenvironments contained more phosphorylated lamin-A/C, resulting in increased degradation.^35,37^ This concept of dynamic remodeling of the lamina is supported by recent work from Guilluy and colleagues, who demonstrated that, in isolated nuclei, recruitment of lamin-A/C to the LINC complex (and nuclear lamina) occurred when dynamic tension was applied via nesprin coated beads.^4^ This response depended on the phosphorylation of emerin, a LINC associated protein that shuttles across the nuclear membrane and plays a role in the cellular mechano-response.^38^ Similarly, in MSCs exposed to dynamic tensile loading, lamin-A/C cortical reorganization was observed within a few days of loading onset.^39^ These changes likely reflect both increased lamin-A/C production coupled with force-induced depletion of the soluble lamin-A/C pool.

While the above studies shed light on the dynamic role of lamin-A/C in nuclear mechano-adaptation, there are almost certainly other factors that regulate this process. For instance, the INM protein LAP2α mediates interactions between transcription factor binding sites on promoters and nucleoplasmic lamin-A/C, as well as playing a key role in th maintenance of the nucleoplasmic lamin pool.^40^ LAP2α also complexes with chromatin through interactions with Barrier-to-Autointegration Factor (BAF), which in turn binds to DNA without sequence specificity.^41^ Similarly, the INM protein Lamin-B Receptor (LBR) binds with both lamin-B and Heterochromatin Protein 1 (HP1) to help shuttle chromatin to the nuclear periphery, and LAP2β binds with both lamin-B and BAF to mediate chromatin tethering.^42,43^ Such interactions function in anchoring chromatin to the nuclear lamina and lead to subsequent increases in nuclear stiffness.^44^ Interestingly, Naetar and coworkers found that epithelial cells from LAP2α-deficient mice contained no nucleoplasmic lamin-A/C, a phenotype which was rescued with re-expression of full length LAP2α.^45^ Studies on stem cells further have shown that loss of nucleoplasmic lamin-A/C during myoblastic differentiation is strongly correlated with a loss of LAP2α, implicating the two as co-requisite binding partners for their nucleoplasmic sequestration.^46^ Furthermore, osmotic stress plays an important role in regulating nuclear structure and function. The mechanical and osmotic properties of the nucleus are intimately coupled, such that osmotic stress on the nucleus can induce dramatic changes in the volume, shape, and mechanics of the nucleus.^47^ For example, hyper-osmotic stress has been shown to reduce nuclear volume and enhance chromatin condensation, with speculation that such changes may represent a direct, biophysical means by which osmotic stresses can regulate intracellular signaling.^48^ Moving forward, it will be important to establish the key regulators of dynamic modeling at the nuclear lamina and the role that mechanical forces play in its regulation.

## Mechano-adaptation in the epigenome

As noted above, mechano-adaptation of the nucleus in response to mechanical cues can occur at the connection to the cytoskeleton and at the level of the nuclear lamina. These mechano-adaptive processes extend to the epigenome as well, where mechanical forces can alter the organization and compaction of chromatin as well as its association with the nuclear lamina (Fig. 1*; Chromatin*). Epigenetics can be broadly defined as the heritable, non-genetic changes which link genotype to phenotype.^49^ The genome is physically compacted and organized within the nucleus as chromatin, the combination of DNA and its tightly associated proteins. Chromatin structure is dictated to a great extent by DNA methylation and the organization of nucleosomes, or strands of DNA wrapped around histone octamers. In mammals, DNA methyltransferases (DNMTs) catalyze methylation of cytosine residues in CpG dinucleotides, with context dependent roles in regulation of chromatin structure and gene expression.^50^ Additionally, amino acid residues in histone sub-units undergo a variety of post translational modifications, influencing the binding affinities of nucleosomes and in turn the spatial organization and transcriptional activity of closely associated DNA.^51^ An additional layer of regulation is conferred by structural proteins, which organize chromatin into three dimensional loops and domains.^52^

Chromatin remodeling evoked by mechanical loading can alter the mechanical properties of the stem cell nucleus. Prior to lineage commitment, ES cells downregulate pluripotency markers and de-condense their chromatin, with concomitant softening of their nuclei.^53^ Chemical modification of chromatin state via condensers (e.g., MgCl_2_ and CaCl_2_) and de-condensers (e.g., Trichostatin A [TSA] and 5-AZA-2′-deoxycytodine [AZA]) stiffened and softened the nucleus, respectively.^53^ Such results support a link between the chromatin condensation state and overall nuclear mechanics, as ES cells lack lamin-A/C (and thus the ability to change nuclear mechanics through regulation of the nuclear lamina). Interestingly, the mode of nuclear mechano-adaptation appears to depend on the level of mechanical input. That is, manipulation of individual isolated nuclei showed that small deformations (<3 μm) drove changes in chromatin remodeling, while larger deformations elicited alterations in lamin-A/C organization, with both responses serving to stiffen the nucleus.^54^ Similarly, chromatin condensation in MSCs induced by 10 minutes of dynamic loading coincided with reduced nuclear deformability,^55^ a time period that was likely too short for alterations in the expression or organization of nuclear lamina components. Further, in these dynamically loaded MSCs, marks of chromatin condensation (H3K27me3) became apparent after only one day of loading, while reorganization of the lamina took several days.^39^ Together, these findings implicate chromatin remodeling in the stiffening of the nucleus in the early response to mechanical stimuli, and suggest that mechano-adaptation of the epigenome can occur more rapidly than mechano-adaptation of the nuclear lamina.

Importantly, the nature, time scale, and degree of force-induced remodeling of the epigenome depend on the specific mode and parameters of the mechanical input. Initial studies in this area showed that dynamic loading causes changes in chromatin condensation, altering mechanical properties of the nucleus. For instance, a single force pulse of 1.25 nN generated by plasma membrane adhered magnetic beads resulted in rapid (< 5s) chromatin de-compaction.^56^ Conversely, tensile loading of MSCs (applied across the entire cell) resulted in chromatin condensation as soon as 10 minutes after the initiation of loading,^55^ an effect which depended on the activity of histone modifying enzymes (i.e., the histone methyltransferase EZH2). Notably, both responses required intact cellular mechanobiological machinery and patent force transfer to the nucleus, as pharmacological disruption of cell contractility abrogated this force-induced chromatin remodeling.^57^

While the above clearly implicates chromatin structure and state as a mechano-responsive element in stem cell nuclei, the precise mechanisms of mechanical regulation of the epigenome remain largely undetermined. Early studies on the mechanical regulation of the epigenome hint at its complex role in governing cell function and differentiation. In vitro, fluid shear stress modulates the activity of histone deacetylases (HDACs) in vascular endothelial cells, with functional implications in their inflammatory and oxidative responses as well as differential expression of genes linked to atherosclerosis^58^ Additionally, physical cues can drive both locus-specific and global epigenetic changes, with corresponding changes in cell pluripotency and program. For instance, in mouse MSCs, oscillatory fluid flow decreased DNA methylation of bone a bone-specific gene promoter (osteopontin), with a corresponding increase in expression of that gene.^59^ Mechanical stimulation via cyclic mechanical stretch also decreased HDAC1 activity in MSCs undergoing osteogenesis, with associated increase in Notch signaling and bone formation.^60^ Additionally, in mouse induced pluripotent stem cells (iPSCs), cell geometry and interactions with the mechanical microenvironment led to globally increased levels of H3 acetylation and methylation, with the magnitude of these changes comparable to that evoked by chemical regulators (valproic acid and Tranyl-cypromine hydrochloride); these changes ultimately improved reprogramming efficiency.^61^ Furthermore, dynamic tensile loading of human epithelial progenitor cells resulted in an emerin and nuclear actin-mediated global reduction in transcription and increase in H3K27me3, as well as a switch from the constitutive heterochromatin marker H3K9me3 to H3K27me3.^62^

These mechanically induced changes in chromatin conformation and anchorage can profoundly impact transcriptional regulation, both directly and indirectly. For instance, it has recently been demonstrated that physical deformation at the cell boundary can percolate through the cell and into the nucleus. In one recent study, large artificial chromosome constructs were inserted into the genome followed by stretch applied through magnetic beads.^2^ Based on tracking of GFP localizations along the insert, the authors were able to demonstrate that perturbations at the cell boundary led directly to physical deformation of the inserted chromatin constructs, and that this deformation enhanced transcriptional activity from that locus. This suggests that direct transfer of force from the extracellular environment to chromatin can alter gene expression. As such, any change in the conformation or physical properties of the epigenome (as occurs with mechano-adaptation of the nucleus) would be expected to modulate this phenomenon. Changes in chromatin density can also alter transcriptional activities in an indirect manner. For instance, when nuclei were treated with MgCl_2_, chromatin rapidly condensed and the nuclei stiffened.^39,54^ When these cells with stiffer nuclei were exposed to stretch, they initiated calcium signaling both at a lower threshold and to a greater extent compared to cells with softer nuclei.^39^ Thus, the physical properties of chromatin, regulated by its condensation state, may act as a mechanical rheostat, adjusting the sensitivity of a cell to physical perturbation.

## Consequences of nuclear mechano-adaptation

Mechano-adaptation of the stem cell nucleus happens across length scales, from the cytoskeleton to the nuclear lamina, down to sub-nuclear structures including chromatin (Fig. 1). These mechano-adaptive events can happen over a short time scales, via force induced alterations in protein assemblies or by changing the activities of key enzymatic modifiers of the substructures. When the inputs driving such processes continue over longer times, a more fundamental and persistent mechano-adaptation can occur via synthesis of new or additional components, and this longer-term adaptation can regulate how subsequent physical signals are interpreted by the cells (Fig. 2). In essence, the culmination of these inputs contributes to the ‘mechanical memory’ of these cells, mediated by mechano-adaptation of the nuclear lamina, its connections, and the state of the epigenome. A growing body of evidence supports this concept. For instance, Yang and colleagues demonstrated that MSCs pre-cultured on stiff substrates for longer periods of time retained a ‘stiff’ phenotype in terms of nuclear localization of YAP and RUNX2, even after transfer to a softer material.^63^ Interestingly, they showed this to be a dose-dependent phenomenon, where culture on stiff substrates for short periods of time resulted in transient activation while longer mechanical dosing resulted in more persistent or near-permanent activation.^63^ Li and coworkers showed the functional implications of such mechanical memory, where MSCs expanded on stiff culture substrates induced a pro-fibrotic program (and increased scar formation in an in vivo model), while those primed on a soft substrate did the opposite.^64^ They further identified expression of the non-coding microRNA miR-21 as one potential mediator of this memory. Results from our group further expand this concept, and suggest that mechanical memory, at least in part, resides in mechano-adaptation of the epigenome. That is, not only does dynamic loading induce chromatin condensation in MSCs, but repeated cycles of loading results in a greater degree of chromatin condensation that persists for longer periods of time after the cessation of loading.^55^ Importantly, the persistence of this chromatin condensation was dependent on epigenetic modifications, as application of a small molecule inhibitor EZH2 (a chromatin remodeler) after condensation resulted in a rapid loss of this mechanical memory. Taken together, these results suggest that classic mechanotransductive signals such as transcription factors, non-coding RNA, and effectors of histone and chromatin modifications are critical drivers and outcomes of mechanical memory; nevertheless, the precise details of their mechanical regulation remain to be determined.

## Summary and conclusions

The experimental evidence discussed in this Perspective outlines our current understanding of mechanical adaptation of the stem cell nucleus. These general principles are not limited to stem cells; in cancer, diseased cells undergo changes in their mechanical and morphological properties, accruing increased cellular contractility and deformability as well as larger and irregularly shaped nuclei^65^ and alterations in the tumor mechanical microenvironment.^66^ Improved understanding of the progression of such physical adaptations of the nucleus in this context of mal-adaptive phenotype transformation could provide new means of mechanobiologically diagnosing and/or targeting the disease.

Regardless of these diverse cellular contexts in which nuclear mechano-adaptation can occur, we highlight in this Perspective how mechanical inputs to stem cells are propagated from the extracellular environment through a patent cytoskeleton to the nucleus (and sub-nuclear structures). Following this, physical signals are, by a variety of mechanisms, transduced to molecular, biochemical, organizational, and structural outputs which in turn adjust the mechanobiological state, serving to adapt cellular machinery to future mechanical input as well as drive functional outcomes. Mechanical memory, then, refers to a specific subset of the cellular mechano-adaptive response where, following a defined mechanical input (i.e., a “mechanical dosage”) which exceeds some threshold, the outputs resulting from the mechanotransductive response persist long past the cessation of the original mechanical cue and continue to influence both the cellular mechano-response as well as functional behavior of the cell. It follows then that mechanically-mediated differentiation is the accumulation of these mechano-adaptive events that are stored first in short-term mechanical memories, then ultimately encoded in long-term cell memories that define stable terminal differentiation. Further explication of these mechano-adaptive and memory storage mechanisms will be required to fully detail the means by which these mechanical signals can first inform stem cell differentiation, and subsequently enforce this commitment to phenotype.
